# Emerging infections: getting ahead of the curve.

**DOI:** 10.3201/eid0101.950101

**Published:** 1995

**Authors:** D Satcher

**Affiliations:** Centers for Disease Control and Prevention, Atlanta, GA 30333, USA. eieditor@cidod1.em.cdc.gov

## Abstract

The early history of infectious diseases was characterized by sudden, unpredictable outbreaks, frequently of epidemic proportion. Scientific advances in the late 19th and early 20th centuries resulted in the prevention and control of many infectious diseases, particularly in industrialized nations. Despite these improvements in health, outbreaks of infectious disease continue to occur, and new infections emerge. Since 1987, the National Academy of Science's Institute of Medicine (IOM) has published three reports that have identified erosion of the public health infrastructure among the factors contributing to new and reemerging infectious diseases. In partnership with many public and private organizations in the United States and abroad, the Centers for Disease Control and Prevention (CDC) has developed a strategic plan that addresses the priorities set forth in the IOM reports and serves as a guide for CDC and its partners to combat emerging microbial threats to health. Laboratory-based surveillance, better communication networks, and improvements in the public health infrastructure are the cornerstones of the strategy. Emerging Infectious Diseases, a new periodical produced by CDC, will serve as a forum for exchange of information about incipient trends in infectious diseases, analysis of factors contributing to disease emergence, and development and implementation of prevention measures.


### Emerging Infections: Getting Ahead of the Curve

**David Satcher, M.D., Ph.D.**
Centers for Disease Control and Prevention, Atlanta, Georgia, USA

*The early history of infectious diseases was characterized by sudden, unpredictable outbreaks, frequently of epidemic proportion. Scientific advances in the late 19th and early 20th centuries resulted in the prevention and control of many infectious diseases, particularly in industrialized nations. Despite these improvements in health, outbreaks of infectious disease continue to occur, and new infections emerge. Since 1987, the National Academy of Science's Institute of Medicine (IOM) has published three reports that have identified erosion of the public health infrastructure among the factors contributing to new and reemerging infectious diseases. In partnership with many public and private organizations in the United States and abroad, the Centers for Disease Control and Prevention (CDC) has developed a strategic plan that addresses the priorities set forth in the IOM reports and serves as a guide for CDC and its partners to combat emerging microbial threats to health. Laboratory-based surveillance, better communication networks, and improvements in the public health infrastructure are the cornerstones of the strategy.* Emerging Infectious Diseases, *a new periodical produced by CDC, will serve as a forum for exchange of information about incipient trends in infectious diseases, analysis of factors contributing to disease emergence, and development and implementation of prevention measures.*

**"Nothing in the world of living things is permanently fixed."**

Hans Zinnser—Rats, Lice and History, 1935

### Early History of Infectious Diseases

Infectious diseases have plagued humans since the dawn of civilization (1-5). The history of these diseases provides a valuable perspective for evaluating current trends. Humans are presumed to have originated in tropical climates and to have been affected by the same parasitic diseases as other primates in these areas. As available supplies of game diminished, early hunters migrated into temperate zones which were free of tropical parasites. Historians speculate that humans were relatively safe from infectious diseases during that period. Later, however, as agriculture began to provide a substantial portion of the human diet, populations stabilized and grew. Eventually, populations reached a size that would support persistent person-to-person spread of infectious microorganisms. With this newly established mode of transmission, infectious diseases soon became widespread. The exact origins of many infectious agents remain obscure, but with the advent of large populations, humans eventually became the established reservoir of many agents. Infected animals and contaminated food and water were additional sources of infectious microorganisms.

Dissemination of infectious diseases intensified as civilizations progressed. Caravans of traders carried new pathogens to unsuspecting and susceptible populations. Explorers and later conquering armies brought infectious microorganisms to new continents. Stowaway rats and other vermin in the holds of ships traveled down the moorings when the ships docked, bringing fleas, lice, and deadly pathogens to a new world. Sporadic epidemics of plague, smallpox, typhus, and measles ravaged cities, decimated armies, and altered the course of history.

### 19th Century Discoveries Lead to Infectious Disease Prevention and Control

Control of many infectious diseases became possible with the pioneering work of Robert Koch and Louis Pasteur and the introduction of the germ theory of disease. With bacteriologic cultivation techniques came the first isolation and identification of etiologic agents; virus cultivation and identification became available some decades later. Reservoirs of microorganisms and their life cycles were identified; the epidemiology and natural history of many infectious diseases were described, and successful control measures were initiated. Water

Address for correspondence: David Satcher, Centers for Disease Control and Prevention, 1600 Clifton Road, Atlanta, GA 30333, USA, fax 404-639-3039, e-mail eideditor@cidod1.em.cdc.gov.

treatment, vector control, and rodent reduction programs followed. By the beginning of the 20th century, the principles of vaccination, established empirically by Edward Jenner more than 100 years earlier, began to be realized in earnest. Antibiotics were discovered, and disinfectants were developed. Collectively, these control measures dramatically decreased the incidence and prevalence of many infectious diseases and their fatality rates. The early part of this century is appropriately regarded as a golden age in public health.

### New and Reemerging Infectious Diseases—A Contemporary Problem

Compared with earlier generations, we possess an enormous scientific base, and the rate of acquisition of new information about infectious diseases is at a historic high. Moreover, thanks in large measure to effective childhood immunization programs, including the President's Childhood Immunization Initiative, many infectious diseases are under control, particularly in the industrialized world. The elimination of smallpox in 1977 stands as a towering achievement in the fight against infectious diseases. However, many infectious diseases have persisted and have displayed a remarkable ability to reemerge after lengthy periods of stability. Therefore, we must be ever mindful of the cyclical nature of disease trends.

A careful review of infectious disease trends shows a fragile equilibrium between humans and infectious microorganisms. Infectious diseases are still broadly endemic and maintain a large reservoir of agents that have the potential for rapid and widespread dissemination. Infectious diseases remain the leading cause of death worldwide, even as the International Code of Diseases places many infectious diseases in other categories. For example, meningitis and cirrhosis are classified as diseases of the nervous system and liver, respectively, and only 17% of deaths attributable to infections are actually included in the codes for parasitic and infectious diseases (6). In the United States, each year, approximately 25% of physician visits are attributable to infectious diseases, with direct and indirect costs, including those for human immunodeficiency virus (HIV) infection and related illnesses, estimated at more than $120 billion (7).

Persons living in tropical climates are still as vulnerable to infectious diseases as their early ancestors were. Each year more than one million children die of malaria in sub-Saharan Africa alone (8); worldwide, approximately 200 million people have schistosomiasis (9), and each year 35-60 million people contract dengue (10). Moreover, infectious diseases and their attendant problems are not confined to tropical climates. For example, an estimated 600,000 cases of pneumonia occur in the United States each year and cause 25,000 to 50,000 deaths (11). More than 10,000 cases of diphtheria have occurred in Russia since 1993 because of inadequate levels of immunization (12). Despite a century of scientific progress, infectious diseases still cause enormous human suffering, deplete scarce resources, impede social and economic development, and contribute to global instability. The potential for even greater dissemination looms as a continuous threat.

Recent outbreaks underscore the potential for the sudden appearance of infectious diseases in currently unaffected populations. In the United States, contamination of the municipal water supply in Milwaukee, Wisconsin, in 1993 resulted in an outbreak of cryptosporidiosis that affected an estimated 400,000 people; approximately 4,400 persons required hospitalization (13). In the 1990s, epidemic cholera reappeared in the Americas, after being absent for nearly a century; from 1991 through June of 1994 more than one million cases and nearly 10,000 deaths were reported (14). During the 1980s, tuberculosis reemerged in the United States after decades of decline, and drug-resistant strains have made its control more difficult (15,16). The increasing prevalence of antibiotic-resistant strains of gonococci, pneumococci, enterococci, and staphylococci portend of other serious treatment and control failures. Many other examples of emerging infections could be given (17,18).

New infectious diseases, often with unknown long-term public health impact, continue to be identified. Table 1 lists major diseases or etiologic agents identified just within the last 20 years (19-41). New agents are regularly added to the list, particularly with the availability of nucleic acid amplification techniques for detecting and identifying otherwise noncultivable microorganisms (40, 42).

In some cases, etiologic agents have been identified as the causes of known diseases or syndromes (e.g., rotavirus, parvovirus, human T-cell lymphotropic viruses I and II (HTLV I/II), and human herpesvirus type 6, (HHV-6); in other cases, diseases became better recognized or defined (e.g., Legionnaires' disease, Lyme disease, human ehrlichiosis). Still others are entirely new: with some parallels to medieval times, a previously unknown and deadly disease, acquired immunodeficiency syndrome (AIDS), originated from uncertain sources in one part of the world and became globally disseminated; this time the disease spread at a rate that would have been unthinkable in medieval times. Clearly, the complete history of infectious diseases remains to be written.

### Getting Ahead of the Curve

Recent disquieting infectious disease trends have not gone unrecognized, and their similarity to earlier disease trends with immense global consequences has not gone unnoticed. Primary responsibility for addressing new and reemerging infectious diseases rests squarely with the custodians of public health. Indeed, the fundamental maxim of public health must guide current prevention programs: the health of the individual is best ensured by maintaining or improving the health of the entire community. Core functions necessary to ensure the health of the public were defined in the National Academy of Science's Institute of Medicine (IOM) report on *The Future of Public Health* (43):

- Assessment of health status, risks, and services
- Development of health policy
- Assurance of quality health services

Surveillance (assessment) is the *sine qua non* of infectious disease prevention programs; however, for surveillance to be effective it must be specific. Consider, for example, surveillance of viral hepatitis. Only after the various agents of viral hepatitis were identified and specific laboratory testing became available was it possible to explain trends in disease prevalence and establish the epidemiologic principles underlying the different modes of transmission. Specific laboratory testing is also the basis of screening programs that ensure the safety of the blood supply against hepatitis B and hepatitis C. Agent-specific surveillance is a critical component of many immunization programs. Vaccines to *Haemophilus influenzae* type b, (Hib), for example, were developed in response to laboratory-based surveillance that identified Hib as a major cause of invasive disease in children. The effectiveness of the Hib vaccination campaign in the United States has been dramatic (Figure 1). Similar approaches will ensure appropriate formulation of other developmental vaccines. Monitoring antibiotic resistance is yet another important example of the value of laboratory-based

**Table 1. Major Etiologic Agents, Infectious Diseases Identified Since 1973***

| Year | Agent | Disease | Reference |
|---|---|---|---|
| 1973 | Rotavirus | Major cause of infantile diarrhea worldwide | 19 |
| 1975 | Parvovirus B19 | Fifth disease; Aplastic crisis in chronic hemolytic anemia | 20 |
| 1976 | *Cryptosporidium parvum* | Acute enterocolitis | 21 |
| 1977 | Ebola virus | Ebola hemorrhagic fever | 22 |
| 1977 | *Legionella pneumophila* | Legionnaires' disease | 23 |
| 1977 | Hantaan virus | Hemorrhagic fever with renal syndrome (HFRS) | 24 |
| 1977 | *Campylobacter* sp. | Enteric pathogens distributed globally | 25 |
| 1980 | Human T-cell lymphotropic virus-I (HTLV I) | T-cell lymphoma—leukemia | 26 |
| 1981 | *Staphylococcus* toxin | Toxic shock syndrome associated with tampon use | 27 |
| 1982 | *Escherichia coli* O157:H7 | Hemorrhagic colitis; hemolytic uremic syndrome | 28 |
| 1982 | HTLV II | Hairy cell leukemia | 29 |
| 1982 | *Borrelia burgdorferi* | Lyme disease | 30 |
| 1983 | Human immunodeficiency virus (HIV) | Acquired immunodeficiency syndrome (AIDS) | 31 |
| 1983 | *Helicobacter pylori* | Gastric ulcers | 32 |
| 1988 | Human herpesvirus-6 (HHV-6) | Roseola subitum | 33 |
| 1989 | *Ehrlichia chaffeensis* | Human ehrlichiosis | 34 |
| 1989 | Hepatitis C | Parenterally transmitted non-A, non-B hepatitis | 35 |
| 1991 | Guanarito virus | Venezuelan hemorrhagic fever | 36 |
| 1992 | *Vibrio cholerae* O139 | New strain associated with epidemic cholera | 37 |
| 1992 | *Bartonella* (= *Rochalimaea*) *henselae* | Cat-scratch disease; bacillary angiomatosis | 38, 39 |
| 1993 | Hantavirus isolates | Hantavirus pulmonary syndrome | 40 |
| 1994 | Sabiá virus | Brazilian hemorrhagic fever | 41 |

*Compiled by CDC staff. Dates of discovery are assigned on the basis of the year the isolation or identification of etiologic agents was reported.

surveillance. Within this context, current discoveries of etiologic agents and diseases (Table 1) are reasons for optimism. The potential for improvements in assessment and prevention of these and other newly discovered diseases is reminiscent of the watershed years of Koch and Pasteur.

We cannot overstate the role of behavioral science in our effort to "get ahead of the curve" with emerging infections. Having the science or laboratory technology to control infectious diseases is not enough, unless we can influence people to behave in ways that minimize the transmission of infections and maximize the efforts of medical interventions. For example, even though HIV/AIDS does not have a vaccine or cure, it is almost entirely preventable. For many people, however, reducing the risk for HIV infection and AIDS requires important changes in lifestyle or behavior. We must use our knowledge of human behavior to help people make lifestyle changes and prevent disease.

Another illustration of the need to use behavior science is the problem of antibiotic resistance. To a great extent, this problem is related to the behavior of both physicians and patients. Physicians continue to use antibiotics inappropriately, and patients continue to demand antibiotic treatment when it is not indicated, for example, for the common cold. As society changes and institutions such as day care centers and prisons become more crowded, the spread of infectious diseases is exacerbated. For homeless and drug-dependent populations, completing a 6- to 9-month course of therapy for tuberculosis is difficult, and the failure to complete the therapy increases the risk for drug-resistant tuberculosis in the community.

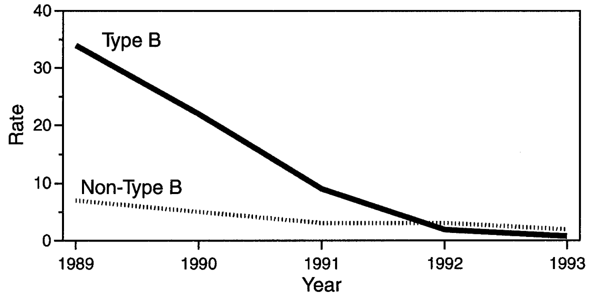

Figure 1. Race-adjusted incidence rate* of *Haemophilus influenzae* type b and non-type b disease detected through laboratory-based surveillance† among children aged <5 years — United States, 1989–1993

* Per 100,000 children aged <5 years.

† The surveillance area population was 10.4 million in four states (three counties in the San Francisco Bay Area, eight counties in metropolitan Atlanta, four counties in Tennessee, and the state of Oklahoma.

Source: CDC. Progress toward elimination of *Haemophilus influenzae* type b disease among infants and children — United States, MMWR 1994;43:144-8.

Microbiologists, infectious disease specialists, and other basic and clinical scientists must collaborate with behavioral scientists in an interdisciplinary effort to prevent and control emerging infections.

*The Future of Public Health* emphasizes the relationship between a sound public health infrastructure and infectious disease prevention programs. Infrastructure improvements must become a national priority: certainly they are among CDC's top priorities. Improvements in infectious disease surveillance are particularly needed (44). Enriching the capacity to respond to urgent threats to health and developing nationwide prevention strategies are also CDC priorities. To combat new and reemerging infectious diseases, CDC, in collaboration with other federal agencies, state and local health departments, academic institutions, professional societies, international organizations, and experts in public health infectious diseases and medical microbiology developed a plan—*Addressing Emerging Infectious Disease Threats: A Prevention Strategy for the United States* (7). The plan has four major goals:

- **Surveillance and Response**—detect, promptly investigate, and monitor emerging pathogens, the diseases they cause, and the factors influencing their emergence
- **Applied research**—integrate laboratory science and epidemiology to optimize public health practice
- **Prevention and control**—enhance communication of public health information about emerging diseases and ensure prompt implementation of prevention strategies
- **Infrastructure**—strengthen local, state, and federal public health infrastructures to support surveillance and implement prevention and control programs

CDC's plan provides a framework for the agency to work collaboratively with its many partners to identify and reverse worrisome trends in infectious diseases.

The need for implementing CDC's plan is urgent, given the extremely dynamic nature of disease trends and the complexity of factors contributing to disease emergence; these were outlined in detail in the 1992 IOM report—*Emerging Infections: Microbial Threats to Health in the United States* (45) and are discussed in a companion article by Stephen S. Morse, Ph.D., in this issue. The IOM report concludes that infectious diseases must be viewed as but one component of a dynamic and complex global ecology, which is shaped and buffeted by technologic, societal, economic, environmental, and demographic changes, not to mention microbial change and adaptation.

Clearly, broader coalitions are needed, and communication must improve if we are to "get ahead of the curve." This new periodical is part of the overall strategy to draw worldwide attention to emerging infections and improve communication. Given the multiplicity of factors contributing to disease emergence, *Emerging Infectious Diseases* (EID) will present relevant concepts from professionals in multiple disciplines and disseminate information about emerging infectious diseases in order to develop and apply ecologically acceptable interventions that will benefit humankind. Prevention and control of new and emerging infectious diseases depend on the participation of scientists and other professionals in the public and private sectors.

CDC will make EID available by print and electronically to facilitate rapid communication. We hope that in the process EID will promote the exchange of infectious disease information through global electronic networks and bulletin boards.

Although there are many similarities between our vulnerability to infectious diseases and that of our ancestors, there is one distinct difference: we have the benefit of extensive scientific knowledge. Ultimately, our success in combatting infectious diseases will depend on how well we use available information. A recent report by the Carnegie Commission "Science, Technology, and Government for a Changing World," provides valuable insight in this regard (46). Commenting on the Earth Summit in Rio de Janeiro in 1992, the report emphasizes the need to shift from the "manifestations of environmental changes in the air, land, water, and plant and animal kingdoms to the causes of those changes." Indeed, the advice of that report challenges us all— "our capacity to generate, integrate, disseminate, and apply knowledge will determine the human prospect in the 21st century."

*Dr. Satcher is director of the Centers for Disease Control and Prevention, Atlanta, Georgia. He was president of Meharry Medical College, Nashville, Tennessee, from 1982 to 1993 and is a former faculty member of the King-Drew Medical Center and the UCLA School of Medicine, Los Angeles, California.*

### References

1. Zinsser H. Rats, lice and history. Boston: Little, Brown, and Company, 1935.
2. Hopkins DR. Princes and peasants: smallpox in history. Chicago: University of Chicago Press, 1983.
3. Bollet AJ. Plagues and poxes. New York: Demos Publications, 1987.
4. Burnet M, White DO. Natural history of infectious disease. London: Cambridge University Press, 1972.
5. McNeill WH. Plagues and peoples. Garden City, New York: Anchor Press/Doubleday, 1976.
6. Bennett JV, Holmberg SD, Rogers MF, Solomon SL. Infectious and parasitic diseases. In: Amler RW, Dull HB, editors. Closing the gap: the burden of unnecessary illness. New York:Oxford University Press, 1987.
7. Centers for Disease Control and Prevention. Addressing emerging infectious disease threats: a prevention strategy for the United States. Atlanta, Georgia: U.S. Department of Health and Human Services, Public Health Service, 1994.
8. World Health Organization. Severe and complicated malaria. Trans R Soc Trop Med Hyg 1990; 84:Supp 2:1-65.
9. World Health Organization. Tropical disease research: progress 1991-92—Eleventh Programme Report of the UNDP/World Bank, WHO Special Programme for Research and Training in Tropical Diseases (TDR). Geneva: World Health Organization, 1993.
10. Gubler DJ. Vector-borne diseases. In: Encyclopedia of the environment. New York: Houghton Mifflin Co., 1994.
11. Marston BJ, Plouffe JF, Breiman RF, et al. Preliminary findings of a community-based pneumonia incidence study. In: Barbaree JM, Breiman RF, Dufour AP, editors. *Legionella*: current status and emerging perspectives. Washington, D.C.; American Society for Microbiology, 1993.
12. Centers for Disease Control and Prevention. Diphtheria outbreak—Russian Federation, 1990-1993. MMWR 1993; 42:840-1, 847.
13. MacKenzie WR, Hoxie NJ, Proctor ME, et al. A massive outbreak in Milwaukee of *Cryptosporidium* infection transmitted through the public water supply. N Engl J Med 1994; 331:161-7.
14. Organizacion Panamericana de la Salud. El colera en las Americas. Informe No. 10; Junio 1994.
15. Centers for Disease Control and Prevention. Expanded tuberculosis surveillance and tuberculosis morbidity—United States, 1993. MMWR 1994; 43:361-6.
16. Centers for Disease Control and Prevention. Multidrug-resistant tuberculosis in a hospital—Jersey City, New Jersey, 1990-1992. MMWR 1994; 43:417-9.
17. Murphy FA. New, emerging, and reemerging infectious diseases. Adv Virus Res 1994; 43: 1-52.
18. Morse SS, editor. Emerging viruses. New York: Oxford University Press, 1993.
19. Bishop RF, Davidson GP, Holmes IH, Ruck BJ. Virus particles in epithelial cells of duodenal mucosa from children with acute non-bacterial gastroenteritis. Lancet 1973; 2:1281-3.
20. Cossart YE, Field AM, Cant B, Widdows D. Parvovirus-like particles in human sera. Lancet 1975; 1:72-3.
21. Nime FA, Burek JD, Page DL, Holscher MA, Yardley JH. Acute enterocolitis in a human being infected with the protozoan *Cryptosporidium*. Gastroenterology 1976; 70: 592-8.
22. Johnson KM, Webb PA, Lange JV, Murphy FA. Isolation and partial characterization of a new virus causing acute haemorrhagic fever in Zaire. Lancet 1977; 1:569-71.

23. McDade JE, Shepard CC, Fraser DW, Tsai TR, Redus MA, Dowdle WR, Laboratory Investigation Team. Legionnaires' disease. 2: Isolation of a bacterium and demonstration of its role in other respiratory disease. N Engl J Med 1977; 297:1197-1203.
24. Lee HW, Lee PW, Johnson KM. Isolation of the etiologic agent of Korean hemorrhagic fever. J Infect Dis 1978; 137:298-308.
25. Skirrow MB. *Campylobacter* enteritis: a "new" disease. Br Med J 1977; 2:9-11.
26. Poiesz BJ, Ruscetti FW, Gazdar AF, Bunn PA, Minna JD, Gallo RC. Detection and isolation of type C retrovirus particles from fresh and cultured lymphocytes of a patient with cutaneous T-cell lymphoma. Proc Natl Acad Sci 1980; 77: 7415-9.
27. Schlievert PM., Shands KN, Gan BB, Schmid GP, Nishimura RD. Identification and characterization of an exotoxin from *Staphylococcus aureus* associated with toxic shock syndrome. J Infect Dis 1981; 143:509-16.
28. Riley LW, Remis RS, Helgerson SD, et al. Hemorrhagic colitis associated with a rare *Escherichia coli* serotype. N Engl J Med 1983; 308:681-5.
29. Kalyanaraman S, Sarangadharan MG, Poiesz B, Ruscetti FW, Gallo RC. Immunological properties of a type C retrovirus isolated from cultured human T-lymphoma cells and comparison to other mammalian retroviruses, J Virol 1981; 38:906-15.
30. Burgdorfer W, Barbour AG, Hayes SF, Benach JL, Grunwaldt E, Davis JP. Lyme disease-a tick-borne spirochetosis?. Science 1982; 216:1317-9.
31. Barré-Sinoussi F, Chermann JC, Rey F, et al. Isolation of a T-lymphotropic retrovirus from a patient at risk for acquired immune deficiency syndrome. Science 1983; 220:868-71.
32. Marshall B. Comment in: Lancet 1983; 1:1273-5.
33. Yamanishi K, Okuno T, Shiraki K, Takahashi M, Kondo T, Asano Y, Kurata T. Identification of human herpesvirus-6 as a causal agent for exanthem subitum. Lancet 1988; 1:1065-7.
34. Dawson JE, Anderson BE, Fishbein DB, Sanchez JL, Goldsmith CS, Wilson KH, Duntley CW. Isolation and characterization of an *Ehrlichia* sp. from a patient diagnosed with human ehrlichiosis. J Clin Microbiol 1991; 29:2741-5.
35. Choo QL, Kuo G, Weiner AJ, Overby LR, Bradley DW, Houghton M. Isolation of a cDNA clone derived from a blood-borne non-A, non-B viral hepatitis genome. Science 1989; 244:359-61.
36. Salas R, de Manzione N, Tesh RB, Rico-Hesse R, Shope RE, Betancourt A, Goday O, Bruzual R, Pacheco ME, Ramos B, Taibo ME, Tamayo JG, Jaimes E, Vasquez C, Araoz, F, Querales J. Venezuelan hemorrhagic fever. Lancet 1991; 338:1033-6.
37. World Health Organization. Epidemic diarrhea due to *Vibrio cholerae* non-01. Wkly Epidemiol Rec 1993; 68:141-2.
38. Regnery RL, Anderson BE, Clarridge JE III, Rodríguez-Barradas MC, Jones DC, Carr JH. Characterization of a novel *Rochalimaea* species, *R. henselae* sp. nov., isolated from blood of a febrile, human immunodeficiency virus-positive patient. J Clin Microbiol 1992; 30:265-74.
39. Welch DF, Pickett DA, Slater LN, Steigerwalt AG, Brenner DJ. *Rochalimaea henselae* sp. nov., a cause of septicemia, bacillary angiomatosis, and parenchymal bacillary peliosis. J Clin Microbiol 1992; 30:275-80.
40. Nichol ST, Spiropoulou CF, Morzunov S, Rollin PE, Ksiazek TG, Feldmann H, Sanchez A, Childs J, Zaki S, Peters, CJ. Genetic identification of a hantavirus associated with an outbreak of acute respiratory illness. Science 1993; 262:914-7.
41. Lisieux T, Coimbra M, Nassar ES, Burattini MN, de Souza LT, Ferreira I, Rocco IM, daRosa AP, Vasconcelos PF, Pinheiro FP, et al. New arenavirus isolated in Brazil. Lancet 1994; 343:391-2.
42. Relman DA, Falkow S, LeBoit PE, Perkocha LA, Min K-W, Welch DF, Slater LN. The organism causing bacillary angiomatosis, peliosis hepatitis, and fever and bacteremia in immunocompromised patients. N Engl J Med 1991; 324:1514.
43. Institute of Medicine. The future of public health. Washington, D.C.: National Academy Press, 1988.
44. Berkelman RL, Bryan RT, Osterholm MT, LeDuc JW, Hughes JM. Infectious disease surveillance: a crumbling foundation. Science 1994; 264: 368-70.
45. Institute of Medicine. Emerging infections: microbial threats to health in the United States. Washington, D.C.: National Academy Press, 1992.
46. Malone TF. The institutions of science and the global prospect: the case of environment. In: Science, technology, and government for a changing world: the concluding report of the Carnegie Commission on Science, Technology, and Government. New York: Carnegie Commission, 1993.
